# Challenging the prokaryotic MGE‐defense origin of eukaryotic RNA editing

**DOI:** 10.1002/mlf2.70084

**Published:** 2026-06-09

**Authors:** Yuange Duan, Qiuhua Xie, Jiajia Yang, Wanzhi Cai, Hu Li

**Affiliations:** ^1^ Department of Entomology and State Key Laboratory of Agricultural and Forestry Biosecurity, MOA Key Lab of Pest Monitoring and Green Management, College of Plant Protection China Agricultural University Beijing China

We recently published an article entitled “On the origin, evolution, and maintenance of RNA editing” in *Nucleic Acids Research* ^1^, responding to a review by Bendich and Rogers in the same journal, entitled “The biological and evolutionary consequences of competition between DNA sequences that benefit the cell and DNA sequences that benefit themselves”^2^. In our response, we raised concerns about their view that eukaryotic RNA editing serves as a mechanism for mobile genetic element (MGE) defense and presented evidence that does not support their theory. Indeed, our commentary deviated slightly from the broader scope of their original article. While their focus was more extensive, we only addressed the issue of RNA editing. Additionally, one may argue that we appeared not to provide substantial evidence to refute Bendich and Rogers' hypothesis that “eukaryotic RNA editing originates from bacterial RNA editing system”^2^. We focused solely on the current behavior of eukaryotic RNA editing and did not address their novel perspective of “prokaryotic origin.”

Here, we will further clarify our position. We demonstrate from both logical and objective perspectives that the conclusion proposed by Bendich and Rogers, that eukaryotic RNA editing originates from prokaryotic RNA editing system, seems untenable. We do not deny the overall quality and elegant writing of their article, but we appeal that reaching a correct conclusion requires not only solid facts/observations but also meticulous logical reasoning. Notably, in this article, we use the neutral term “bacterial/prokaryotic RNA editing origin” to describe Bendich and Rogers' view. In their original article, although they did not explicitly propose a direct phage restriction by an adenosine deaminase acting on RNA (RADAR)‐to‐eukaryote trajectory, their wording implies this interpretation because the only bacterial/prokaryotic RNA editing system mentioned in connection with anti‐MGE functions is the RADAR (a two‐gene cassette) defense island^2^. Nevertheless, to avoid a potential straw‐man framing, we describe their position more generally as the “broad prokaryotic‐origin hypothesis” and try to cite the “prokaryotic/bacterial RNA editing system” instead of the “RADAR defense island.”

## THE FOCUS ON RNA EDITING DOES NOT DENY THE MGE‐DEFENSE ROLE OF OTHER MECHANISMS

Bendich and Rogers proposed several ways by which the host and MGE compete for genome occupancy, such as rDNA invasion, intron proliferation, repetitive DNA expansion, epigenetic silencing, RNA editing, and recombination. All ways combine to produce the end result: some fraction of the genome is devoted to the survival and reproduction of the host, and some fraction represents the parasitic MGEs. They contend that “the competition between DNA sequences that promote the survival of the organism and those that prioritize their own propagation plays a dominant role in shaping genetics, biology, and evolution”^2^. This conclusion is definitely correct. However, our subsequent response only commented on one of their ways (RNA editing) because they only mentioned our earlier work^3^ in a single sentence regarding RNA editing^1^. Other mechanisms discussed in their article are not within our expertise and we do not deny the MGE‐defense role of those mechanisms. Nevertheless, if our narrow focus has misled any readers into believing that Bendich and Rogers' article only addresses RNA editing, we apologize and would like to reaffirm that RNA editing is just one of the many mechanisms that they proposed^2^.

In our previous response, we already discussed how RNA editing in extant eukaryotes interacts with transposable elements (TEs)^1^. However, our discussion was based on the premise that adenosine deaminase acting on RNA (ADAR)‐mediated RNA editing was a metazoan invention, which ignored Bendich and Rogers' key argument that “eukaryotic RNA editing for MGE‐defense originates from prokaryotes.” Here, we will discuss this “prokaryotic origin” issue.

## THE AMBIGUOUS MGE‐DEFENSE ROLE OF RNA EDITING IN EUKARYOTES

The premise for assessing whether a conclusion is reasonable is that definitions must be clear. To explore whether “eukaryotic RNA editing for MGE‐defense originates from prokaryotes,” we must first confirm whether eukaryotic RNA editing truly serves as a mechanism for MGE defense. In the description by Bendich and Rogers, the definition of MGE is unclear in that part, as multiple MGE‐defense mechanisms are discussed in a conflated manner^2^. If MGE refers to exogenous viruses, then this conclusion is at least partially correct in animals, as ADAR is well known for interacting with viruses^4^. However, this interaction does not imply that ADAR directly eliminates viruses, nor does ADAR exert selective pressure on them. Studies have indicated that ADAR‐mediated RNA editing actually both defends against viruses and promotes viral spread. For example, editing of RNA viruses provides a source of viral mutation, which, to some extent, accelerates viral escape from immunity or the emergence of drug resistance^5^. Given this dual function, some researchers have advocated for not distinguishing between the antiviral and virus‐promoting roles of RNA editing, but instead considering this binary function as different stages of the same process^4^.

**Figure 1:**
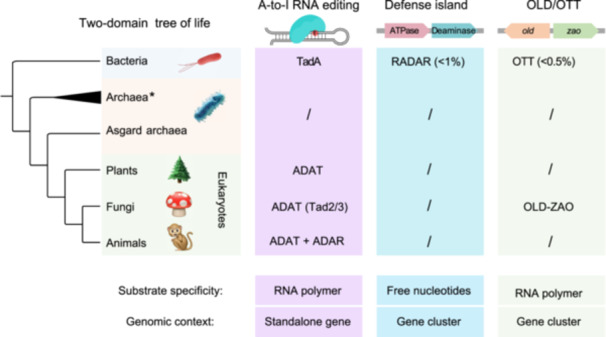
Unscaled phylogenetic relationships of the main clades of the “two‐domain tree of life” and the presence/absence of different systems. The manually drawn phylogenetic tree is inferred from the theory of the “two‐domain tree of life”^9^. The evolution of the A‐to‐I RNA editing system and RADAR and OLD/OTT defense systems is displayed. Asterisk * shows that the labeled Archaea lineage is not a monophyly. Other unlabeled branches are all monophyly. The presence or absence of editing enzymes or systems in the genome of each lineage is indicated. The bottom part describes the substrate and genomic features of the different editing systems. Prokaryotes include both the bacteria and the archaea lineages. ADAR, adenosine deaminase acting on RNA; ADAT, adenosine deaminase acting on tRNA; A‐to‐I, adenosine‐to‐inosine; OLD, OTT_1508‐like deaminase; Tad, tRNA‐specific adenosine deaminase; ZAO, zinc fingers adjacent to OLD.

Fungi possess two currently recognized A‐to‐I RNA editing systems: the OLD–ZAO system (OLD stands for OTT_1508‐like deaminase and ZAO stands for zinc fingers adjacent to OLD)^6^, ^7^ and the Tad2/3 system (Tad stands for tRNA‐specific adenosine deaminase)^8^ (Figure 1). While the former is partially associated with defense functions in certain contexts, as discussed below, the latter appears to be unrelated to MGE defense. In *Neurospora crassa*, A‐to‐I RNA editing mediated by OLD causes the transcription factor ZAO to undergo stop‐codon read‐through, and the extended version of the ZAO protein triggers downstream responses to viral infection^6^. First, the antiviral role of RNA editing is indirect and only represents a few editing sites. This cannot be regarded as a general trend. For instance, we cannot assume that the presence of every transcription factor is for antiviral defense simply because a particular transcription factor activates an antiviral pathway. Second, the generality of this mechanism across different fungal species is unknown. Thus, the virus‐defense role of RNA editing in fungi appears to be weak. In contrast, in plants, the dominant C‐to‐U RNA editing is entirely unrelated to viruses^1^.

We believe that more readers might interpret the term MGE in Bendich and Rogers' article (the section discussing RNA editing) as TEs in the genome. How to define whether RNA editing in eukaryotes performs a TE‐defense or anti‐TE role? This produces a second layer of ambiguity. In our previous article, we clarified that the direct literal meaning of anti‐TEs is to prevent TEs from amplifying within the genome^1^. However, current theories suggest that a shared feature of RNA editing in metazoans is the prevention of immune responses triggered by TE‐derived double‐stranded RNA (dsRNA)^10^. This, in fact, reflects a tolerance for TE amplification, as the expression and expansion of TEs would normally be eliminated due to immune responses, and yet, RNA editing reduces this purifying selection^1^. This evolutionary consequence literally deviates from “TE defense.”

Therefore, in Bendich and Rogers' MGE‐defense theory, both the definitions of “MGE” and “defense” seem ambiguous. Even when considering various interpretations, the MGE‐defense theory is only partially valid in animals, and has only been supported by indirect case studies in fungi, where specific RNA editing sites ultimately led to antiviral responses, rather than a contribution from the entire RNA editing system. Even if an MGE‐defense role is accepted for RNA editing in certain lineages, inferring an ancestral origin for this function requires a plausible evolutionary connection among the relevant RNA editing systems in bacteria, archaea, and eukaryotes. Thus, this theory cannot be generalized to all eukaryotes.

## EXTANT BACTERIA ARE NOT THE ANCESTORS OF EXTANT EUKARYOTES

Since the time of Darwin, evolutionary biology has emphasized that different extant organisms share a common ancestor, and evolution should not be understood as the transition from one existing organism (e.g., monkey) to another (e.g., human). In bacteria, different RNA editing systems like RADAR^11^ and OTT exist^12^ (Figure 1). Bendich and Rogers proposed the idea that RNA editing in eukaryotes represents an attempt to defend against MGEs, and that this defense mechanism originated in prokaryotes^2^. According to the definition in Bendich and Rogers' article, prokaryotes include both bacteria and archaea^2^. However, the authors did not mention the existence of the RADAR or OTT system in archaea, at least reflected by the references cited in Bendich and Rogers' original article^11^. Then, the absence of RADAR in archaea has been further confirmed by recent large‐scale comparative genomic studies^13^. Therefore, the discussion of the prokaryotic RADAR or OTT system is confined solely to bacteria (Figure 1). However, as we will discuss below, while the term “prokaryotic ancestors of eukaryotes” used by Bendich and Rogers^2^ is acceptable when “prokaryotes” encompasses both bacteria and archaea, it becomes misleading if it refers only to bacteria. Extant bacteria are not the ancestors of extant eukaryotes, rendering a direct bacteria‐to‐eukaryote evolutionary trajectory untenable (Figure 1).

Here is a straightforward phylogenetic tree that the union of eukaryotes and archaea forms a sister group with bacteria, thus constituting the two‐domain of life (Figure 1). This phylogeny is currently gaining significant recognition, and we do not aim to enumerate all relevant references here^9^. Therefore, our argument is clear. We can only hypothesize that such a mechanism existed at the eukaryotic ancestor node if both bacteria and archaea show consistent mechanisms, in a parsimonious way. At present, however, the evidence does not support Bendich and Rogers' statement of “prokaryotic (bacterial) ancestors of eukaryotes.”

Indeed, we acknowledge that the common ancestor of bacteria, archaea, and eukaryotes might have been some form of simple cellular organism, but it should not simply be referred to as bacteria. For example, the common ancestor of mammals, reptiles, and birds can be referred to as an amniote, but it cannot be classified as any one of the extant taxa. Likewise, the common ancestor of mammals and stem‐mammal reptiles is designated with a distinct term, synapsids, rather than being called either mammals or reptiles^14^. Nevertheless, it is important to emphasize a key distinction: whereas terms such as human and monkey refer to extant taxa, prokaryote and eukaryote denote cellular types that, by definition, encompass both extinct and extant forms.

## THE BACTERIAL DEFENSE ISLAND IS NOT HOMOLOGOUS TO RNA EDITING SYSTEMS IN EUKARYOTES

Mechanistically, the bacterial defense island is not homologous to RNA editing systems in eukaryotes. Even if we assume that the evidence for RNA editing as an MGE‐defense mechanism is “very strong” in bacteria (which is not completely true, as we discuss in the next section), extending this conclusion to eukaryotic RNA editing still leads to the following issues.

First, these systems have distinct evolutionary origins. The bacterial defense island RADAR system does not exist in eukaryotes^11^. The bacterial OTT editing system is structurally similar but functionally divergent from the fungal OLD editing system, and is not found in other eukaryotic lineages either^6^. In contrast, eukaryotic RNA editing systems are diverse and should not be pooled as a whole when discussing their origin. In animals, adenosine‐to‐inosine (A‐to‐I) RNA editing is carried out by ADAR (for mRNA editing) and adenosine deaminase acting on tRNA (ADAT) (for tRNA editing)^15^, while in fungi and plants, ADAT primarily mediates tRNA editing, with some fungi evolving mRNA editing^16^. Therefore, ADAT represents the ancestral state of A‐to‐I RNA editing enzymes in eukaryotes (Figure 1). ADAR then experienced gains and losses in different animal lineages, where insects lost ADAR1^17^ and mammals obtained an inactive ADAR3^15^. In addition, plants have evolved pentatricopeptide repeat (PPR)‐mediated cytosine‐to‐uridine (C‐to‐U) RNA editing in mitochondria and chloroplasts^18^, and animals also use apolipoprotein B mRNA editing enzymes, the catalytic polypeptide‐like (APOBEC) family for C‐to‐U RNA editing^19^, which clearly have different origins and mechanisms. Moreover, apart from these base modifications, there are also indel type of RNA editing in *Trypanosoma* ^20^. In fact, the term “RNA editing” among these mechanisms only represents a linguistic analogy rather than an evolutionary homology. Researchers may collectively refer to these mechanisms as “eukaryotic RNA editing” in the title or abstract of a review^21^, but when discussing specific mechanisms, “eukaryotic RNA editing” seems overgeneralized. Instead, researchers usually specify whether it is A‐to‐I or C‐to‐U RNA editing^21^, or further clarify whether it refers to mRNA or tRNA editing. This remarkable diversity of mutually unrelated eukaryotic RNA editing systems strongly suggests multiple independent evolutionary origins, which is incompatible with a single inheritance event from prokaryotes. This concern about the overgeneralization of eukaryotic RNA editing has been implicitly indicated in our previous response^1^.

In fact, defense‐related activity is observed in ADAR of particular animals and the fungal OLD‐ZAO system^6^, whereas other eukaryotic editors, such as ADAT‐ and PPR‐type enzymes, lack clear MGE‐defense roles^1^. Then, previous research has indicated that the deaminase structural fold, as well as several eukaryotic editing and mutagenic deaminase families, can ultimately be traced back to bacterial toxin‐related systems^22^. This connection supports a bacterial ancestry for the fold and certain enzymatic functions, but it does not necessarily suggest a single vertical transmission event at the base of eukaryotes, nor a universally conserved defense role among all eukaryotic RNA editing machineries.

In archaea, tRNA I34 does not exist, meaning that A34 is not edited and no archaeal homolog of ADAT or TadA was found^23^. In bacteria, the factor most closely associated with eukaryotic ADAR/ADAT is TadA, not RADAR or OTT (Figure 1). This conclusion is well established. Some literature directly states that “RADAR also likely represents the only RNA A‐to‐I modifying enzyme that does not belong to ADAR/ADAT families”^24^, which clearly indicates that RADAR is distinct from the eukaryotic RNA editing system. However, bacterial TadA has not been reported to be related to MGE defense. At the very least, the work cited by Bendich and Rogers, on TadA, is unrelated to MGE defense^25^. Therefore, drawing an analogy between the bacterial RNA editing system and the eukaryotic RNA editing system seems inappropriate, as they lack homology, and the bacterial state cannot be considered as the ancestral state for eukaryotes (Figure 1).

Here, we further provide a highly illustrative analogy. The wings of insects and birds are not homologous in terms of development, and there is no ancestor–descendant relationship between these two lineages (this “irrelevance” is similar to the phylogenetic relationship between bacteria and eukaryotes). Due to these facts, we cannot conclude that the initial origin of bird wings was aimed for high‐frequency sound production simply because insect wings are capable of producing high‐frequency sounds. This conclusion is not only untenable within the logical framework of phylogenetic trees but it also disregards the important fact that the two types of wings have different origins. One direct consequence of this “different origin” is that their performances and functions are distinct, and most bird wings are incapable of vibrating at the high frequencies seen in insects. Thus, overgeneralization might undermine proper classification and grouping, and discussing unrelated phenomena together sometimes makes it difficult to derive accurate patterns.

In addition to the different origins of the bacterial editing system and eukaryotic ADAR, the second point that we emphasize is that the RADAR system used by bacteria for MGE defense is able to edit free nucleotides, converting them from A to I (Figure 1). For example, ATP and dATP are, respectively, converted into inosine triphosphate (ITP) and deoxy‐ITP (dITP)^24^. This suggests that RADAR even potentially has deamination effects on DNA, which fundamentally distinguishes it from RNA editing mediated by bacterial TadA or eukaryotic ADAT/ADAR. Again, here, the notion of “editing” in the bacterial RADAR system and in the eukaryotic ADAT/ADAR system merely represents a linguistic resemblance rather than a true molecular homology.

In our view, the bacterial editing system for MGE defense does not resemble the traditional RNA editing mechanism in eukaryotes, as the substrates and products are functionally different. However, in the Bendich and Rogers' article, they were treated as the same evolutionary system^2^, and this analogy seems lack empirical support, overgeneralizing things that are fundamentally unrelated.

## RADAR AND OTT EDITING SYSTEMS ARE ONLY FOUND IN <1% BACTERIAL SPECIES

A more critical piece of evidence is that the proportion of bacteria with the RADAR system is extremely low. According to the prediction made by the online tool DefenseFinder^13^, “among the 22,818 complete genomes of RefSeq, the RADAR is detected in 135 genomes (0.59%)” (https://defensefinder.mdmlab.fr/wiki/defense-systems/radar). No archaeal genomes possess RADAR. This is sufficient to demonstrate that the RADAR system is a derived trait in bacteria, rather than being present in the ancestor of bacteria, and it cannot be considered the ancestral state of eukaryotes (Figure 1). Similarly, the OTT_1508 system is only found in less than 0.5% of the bacterial genomes^12^, which also indicates that OTT is not universally present among prokaryotes and therefore is unlikely to have been inherited by the eukaryotic ancestor and subsequently maintained in eukaryotic lineages (Figure 1).

In summary, we conclude that the prokaryotic origin hypothesis is not supported by current data. Extant bacteria are not the ancestors of extant eukaryotes. Then, the bacterial RADAR defense system is evolutionarily unrelated to the diverse RNA editing systems in eukaryotes. The bacterial OTT defense system is structurally homologous to but functionally diverged from the fungal OLD system. More importantly, the RADAR or OTT system only exists in less than 1% of bacteria, suggesting that these mechanisms are derived traits rather than ancestral traits in bacteria.

A limitation of our current argument is that the absence of archaeal RNA editing systems is based on currently available genomes, and therefore should not be interpreted as definitive evidence of complete absence. This issue is especially important for Asgard archaea, given their close evolutionary relationship to eukaryotes. Future studies could therefore perform homolog search for deaminase‐related systems in expanded archaeal datasets, ideally combining sequence searching, structural prediction, and transcriptomic evidence. Such work will help determine whether any archaeal editing‐like systems exist and whether they have evolutionary relevance to eukaryotic RNA editing.

## References

[1] Duan Y , Cao Q , Xie Q , Ma L , Cai W , Li H . On the origin, evolution, and maintenance of RNA editing. Nucleic Acids Res. 2025;53:gkaf947. 40990244 10.1093/nar/gkaf947 PMC12458077

[2] Bendich AJ , Rogers SO . The biological and evolutionary consequences of competition between DNA sequences that benefit the cell and DNA sequences that benefit themselves. Nucleic Acids Res. 2025;53:gkaf589. 40626553 10.1093/nar/gkaf589 PMC12235515

[3] Xie Q , Duan Y . An ultimate question for functional A‐to‐I mRNA editing: why not a genomic G? J Mol Evol. 2025;93:185–192. 39964487 10.1007/s00239-025-10238-8

[4] Piontkivska H , Wales‐Mcgrath B , Miyamoto M , Wayne ML . ADAR editing in viruses: an evolutionary force to reckon with. Genome Biol Evol. 2021;13:evab240. 34694399 10.1093/gbe/evab240 PMC8586724

[5] Patterson JB , Cornu TI , Redwine J , Dales S , Lewicki H , Holz A , et al. Evidence that the hypermutated M protein of a subacute sclerosing panencephalitis measles virus actively contributes to the chronic progressive CNS disease. Virology. 2001;291:215–225. 11878891 10.1006/viro.2001.1182

[6] Honda S , Yokoyama A , Suzuki N . RNA editing of genomic neighbors controls antiviral response in fungi. Cell Host Microbe. 2025;33:545–559. 40132592 10.1016/j.chom.2025.02.016

[7] Du Y , Jiang D , Liu H . RNA editing system: balancing altruistic antiviral defense and fitness trade‐offs in fungi. mLife. 2025;4:597–601. 10.1002/mlf2.70057 PMC12754623 41479410

[8] Feng C , Xin K , Du Y , Zou J , Xing X , Xiu Q , et al. Unveiling the A‐to‐I mRNA editing machinery and its regulation and evolution in fungi. Nat Commun. 2024;15:3934. 38729938 10.1038/s41467-024-48336-8 PMC11087585

[9] Zhang J , Feng X , Li M , Liu Y , Liu M , Hou LJ , et al. Deep origin of eukaryotes outside Heimdallarchaeia within Asgardarchaeota. Nature. 2025;642:990–998. 40335687 10.1038/s41586-025-08955-7 PMC12222021

[10] Liddicoat BJ , Piskol R , Chalk AM , Ramaswami G , Higuchi M , Hartner JC , et al. RNA editing by ADAR1 prevents MDA5 sensing of endogenous dsRNA as nonself. Science. 2015;349:1115–1120. 26275108 10.1126/science.aac7049 PMC5444807

[11] Gao L , Altae‐Tran H , Böhning F , Makarova KS , Segel M , Schmid‐Burgk JL , et al. Diverse enzymatic activities mediate antiviral immunity in prokaryotes. Science. 2020;369:1077–1084. 32855333 10.1126/science.aba0372 PMC7985843

[12] Danov A , Segev O , Bograd A , Ben Eliyahu Y , Dotan N , Kaplan T , et al. Toxinome—the bacterial protein toxin database. mBio. 2024;15:e0191123. 38117054 10.1128/mbio.01911-23 PMC10790787

[13] Tesson F , Hervé A , Mordret E , Touchon M , d'Humières C , Cury J , et al. Systematic and quantitative view of the antiviral arsenal of prokaryotes. Nat Commun. 2022;13:2561. 35538097 10.1038/s41467-022-30269-9 PMC9090908

[14] Weisbecker V . Evolution: bend it like basal synapsids. Curr Biol. 2021;31:R437–R439. 33974869 10.1016/j.cub.2021.03.017

[15] Savva YA , Rieder LE , Reenan RA . The ADAR protein family. Genome Biol. 2012;13:252. 23273215 10.1186/gb-2012-13-12-252 PMC3580408

[16] Duan Y , Li H , Cai W . Adaptation of A‐to‐I RNA editing in bacteria, fungi, and animals. Front Microbiol. 2023;14:1204080. 37293227 10.3389/fmicb.2023.1204080 PMC10244538

[17] Keegan LP , Mcgurk L , Palavicini JP , Brindle J , Paro S , Li X , et al. Functional conservation in human and *Drosophila* of metazoan ADAR2 involved in RNA editing: loss of ADAR1 in insects. Nucleic Acids Res. 2011;39:7249–7262. 21622951 10.1093/nar/gkr423 PMC3167634

[18] Duan Y , Cai W , Li H . Chloroplast C‐to‐U RNA editing in vascular plants is adaptive due to its restorative effect: testing the restorative hypothesis. RNA. 2023;29:141–152. 36649983 10.1261/rna.079450.122 PMC9891260

[19] Liu Z , Zhang J . Human C‐to‐U coding RNA editing is largely nonadaptive. Mol Biol Evol. 2018;35:963–969. 29385526 10.1093/molbev/msy011 PMC6455930

[20] Carnes J , Trotter JR , Peltan A , Fleck M , Stuart K . RNA editing in *Trypanosoma brucei* requires three different editosomes. Mol Cell Biol. 2008;28:122–130. 17954557 10.1128/MCB.01374-07 PMC2223309

[21] Zhang J , Xu C . Gene product diversity: adaptive or not? TIG. 2022;38:1112–1122. 35641344 10.1016/j.tig.2022.05.002 PMC9560964

[22] Iyer LM , Zhang D , Rogozin IB , Aravind L . Evolution of the deaminase fold and multiple origins of eukaryotic editing and mutagenic nucleic acid deaminases from bacterial toxin systems. Nucleic Acids Res. 2011;39:9473–9497. 21890906 10.1093/nar/gkr691 PMC3239186

[23] Grosjean H , De crécy‐Lagard V , Marck C . Deciphering synonymous codons in the three domains of life: co‐evolution with specific tRNA modification enzymes. FEBS Lett. 2010;584:252–264. 19931533 10.1016/j.febslet.2009.11.052

[24] Gao Y , Luo X , Li P , Li Z , Ye F , Liu S , et al. Molecular basis of RADAR anti‐phage supramolecular assemblies. Cell. 2023;186:999–1012.e20. 36764292 10.1016/j.cell.2023.01.026

[25] Bar‐Yaacov D , Mordret E , Towers R , Biniashvili T , Soyris C , Schwartz S , et al. RNA editing in bacteria recodes multiple proteins and regulates an evolutionarily conserved toxin‐antitoxin system. Genome Res. 2017;27:1696–1703. 28864459 10.1101/gr.222760.117 PMC5630033

